# Supplementary material for: Correlation between Neutrophil Extracellular Traps (NETs) Expression and Primary Graft Dysfunction Following Human Lung Transplantation
Source: Cells. 2022 Oct 29;11(21):3420. doi: 10.3390/cells11213420 (PMC9656095; doi:10.3390/cells11213420)
Supplement: Supplementary file 1 [file cells-11-03420-s001.zip › cells-1903234-supplementary.pdf]

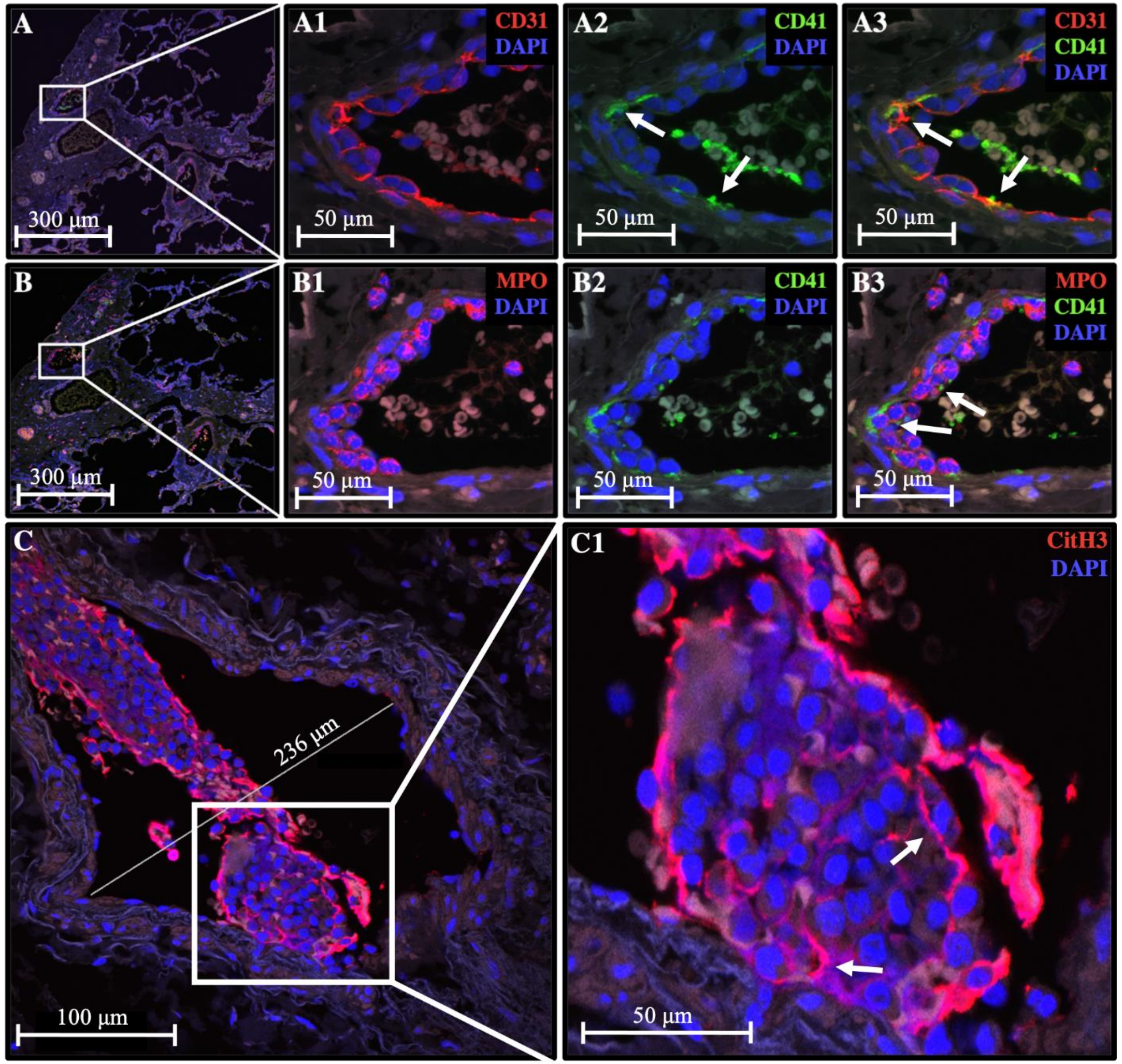


**Figure S1**. Representative confocal microscopy imaging of neutrophil-platelet and NETs endothelium interactions in a lung graft prior to transplant into a recipient who subsequently developed PGD3. In Figure A, platelets adhered onto and underneath the endothelium (white arrows, *A2* and *A3*). In Figure B, numerous neutrophil-neutrophil (*B1*) and neutrophil-platelet (white arrows, *B3*) interactions were observed. In Figure C, the presence of NETs (CitH3; white arrows) in a neutrophilic thrombus was noted. CD31, endothelium; CD41, platelets; CitH3, NETs; MPO, neutrophils; DAPI, dsDNA. Original magnification, x240.

**Table S1.** Donors and recipients' levels or organ biomarkers

| | All patients (n = 36) | non-PGD3 (n = 27) | PGD3 (n = 9) | *P* value |
|---|---|---|---|---|
| ***Donors*** | | | | |
| Troponin I level (ng/mL) | 53 (0-249) | 10 (0-87) | 280 (39-726) | 0.019 |
| Alanine aminotransferase (U/L) | 45 (20-94) | 30 (19-94) | 54 (26-91) | 0.382 |
| Serum creatinine (mmol/L) | 65 (44-86) | 59 (44-78) | 123 (46 -386) | 0.107 |
| Lactic acid (mmol/L) | N.A. | N.A. | N.A. | N.A. |
| ***Recipients*** | | | | |
| Troponin I levels (ng/mL)[a] | | | | |
| 3hrs after 2nd lung implantation | 455 [397-522] | 398 [346-457] | 659 [483-898] | 0.140 |
| Day 1 | 534 [459-621] | 526 [435-634] | 586 [486-707] | 0.684 |
| Day 2 | 396 [340-461] | 389 [322-470] | 423 [347-516] | 0.758 |
| Day 3 | 315 [273-364] | 312 [261-373] | 337 [277-409] | 0.768 |
| Alanine aminotransferase (U/L)[a] | | | | |
| 3hrs after 2nd lung implantation | 20.2 [17.0-24.0] | 14.5 [13.0-16.1] | 54.2 [33.7-87.3] | 0.008 |
| Day 1 | 30.9 [25.6-37.2] | 22.3 [19.6-25.4] | 77.1 [46.8-126.9] | 0.017 |
| Day 2 | 31.5 [26.1-37.9] | 22.3 [19.6-25.3] | 81.7 [50.6-131.9] | 0.010 |
| Day 3 | 35.6 [29.8-42.7] | 24.6 [21.9-27.7] | 96.8 [52.1-150.7] | 0.003 |
| Serum creatinine (mmol/L)[a] | | | | |
| 3hrs after 2nd lung implantation | 70.0 [66.8-73.4] | 66.0 [63.1-68.7] | 83.9 [74.8-94.2] | 0.050 |
| Day 1 | 85.6 [80.4-91.2] | 80.6 [75.0-87.0] | 101.7 [92.0-112.6] | 0.068 |
| Day 2 | 80.9 [74.6-87.7] | 77.5 [69.9-85.5] | 92.7 [83.9-102.3] | 0.204 |
| Day 3 | 73.6 [68.1-79.5] | 70.1 [63.6-76.6] | 86.0 [76.4-96.9] | 0.167 |
| Lactic acid (mmol/L)[a] | | | | |
| 3hrs after 2nd lung implantation | 2.53 [2.28-2.82] | 2.11 [1.92-2.32] | 4.39 [3.48-5.52] | 0.004 |
| Day 1 | 1.91 [1.73-2.11] | 1.57 [1.48-1.67] | 3.41 [2.60-4.47] | 0.006 |
| Day 2 | 1.48 [1.36-1.60] | 1.42 [1.33-1.52] | 1.70 [1.33-2.17] | 0.482 |
| Day 3 | 1.39 [1.26-1.53] | 1.22 [1.10-1.35] | 2.02 [1.65-2.48] | 0.030 |

Data are presented as frequencies (%), means ± standard deviation, medians (IQR) or as [a]estimates [95%CI] from mixed-effect analysis where appropriate. N.A., not available.
